# Immuno-hybrid algorithm: a novel hybrid approach for GRN reconstruction

**DOI:** 10.1007/s13205-016-0536-1

**Published:** 2016-10-14

**Authors:** A. S. Jereesh, V. K. Govindan

**Affiliations:** 1 Department of Computer Science, Cochin University of Science and Technology, Cochin, Kerala India; 2 Department of Computer Science and Engineering, Indian Institute of Information Technology Pala, Kottayam, Kerala India

**Keywords:** Clonal selection algorithm, Immuno-hybrid algorithm, Optimization algorithm, Gene regulatory network, DNA microarray, BFGS Quasi-Newton

## Abstract

Bio-inspired algorithms are widely used to optimize the model parameters of GRN. In this paper, focus is given to develop improvised versions of bio-inspired algorithm for the specific problem of reconstruction of gene regulatory network. The approach is applied to the data set that was developed by the DNA microarray technology through biological experiments in the lab. This paper introduced a novel hybrid method, which combines the clonal selection algorithm and BFGS Quasi-Newton algorithm. The proposed approach implemented for real world *E. coli* data set and identified most of the relations. The results are also compared with the existing methods and proven to be efficient.

## Introduction

It has been seen that, even though the biological systems are very complex, they exhibit very sophisticated behavior in the evolution with time and there are many regularities that have been observed, which leads to the scientific understanding of the biological processes and the behavior. Identification of the relationships between genes leads to better understanding of the complexities of the biological systems at molecular level. GRN is a network of interrelated genes participating in a biological process. Identification of the gene network provides an insight into the important genes that are participating in a bio-chemical process. The major applications of the gene regulatory network include identification of network topology, identification of central node in the network, identification of the sub networks, mutual dependency between genes and dependency between different biological conditions from a computational viewpoint. Recognition of central gene or gene set can be used to synthesize better drugs for the inhibition or activation of the biological processes and the methodology can be extended to drug design or to study the direct drug delivery mechanisms.

This topic of research has been very active for the last one decade. Various researchers have suggested techniques attempting to solve this problem with faster convergence and accuracy. Boolean network is a network of variables whose values are true or false. A gene network can be defined in terms of Boolean network as *G*(*V*, *E*) with a set of states *X* = {*x*_*i*_|*i* = 1…*n*} due to a set of Boolean operations: *B* = {*b*_*i*_|*i* = 1,…,*n*}; *x*_*i*_ (*t* + 1) = *b*_*i*_(*x*_*i*1_,…,*x*_in_) where, *x*_*ij*_ denotes the states of the nodes connected to vertex *i*; ‘*n*’ denotes the number of genes involved in the situation. Kauffman (1969) introduced Boolean network that consists of binary state variables for the construction of gene network. After this a number of Boolean methods were proposed by the researchers (Liang et al. 1998; Akutsu et al. 1999; Maucher et al. 2011). Due to the complexity of the biological systems, the data obtained through the experiments are uncertain and incomplete. The exact modeling of gene regulatory network is a difficult problem. It is possible to represent a relation with more than one function. To solve this problem an advancement of Boolean network is introduced, namely, probabilistic Boolean network. Based on this concept (Shmulevich et al. 2002; Liang and Han 2012) proposed methods for reconstruction of GRN. For all the Boolean models, binary states of the genes represent whether a gene is present or not in the gene network, but do not indicate the extent of its participation in the relation. This leads to lower accuracy in predictions.

Bayesian network is a probabilistic directed acyclic graph model based on the ideas proposed by Bayes et al. (1763). Joint probability distribution is used in the calculation of relationships in Bayesian network. Based on this modeling several standard methods are introduced (Yang et al. 2011; Tan and Mohamad 2012; Dondelinger et al. 2012). Gene network interactions are cyclic and non-linearly complex. Therefore, Bayesian networks may fail if such condition happens.

Artificial neural network is a tool for predicting gene network used by several researchers. Based on the recurrent neural network lots of works have been produced by the researchers (Vohradský 2001; Lee and Yang 2008; Noman et al. 2013). Major disadvantage of the neural network model is the increased complexity with respect to number of genes. Support vector machine is another machine-learning tool for predicting GRN (Kimura et al. 2009).

Reconstruction of gene network using the differential equation model is a popular approach. Apart from the co-expression models, differential equation models have the capability to describe the dynamic behavior of the biological system. S-system (Savageau 2000) is a well-accepted mathematical model for the chemical reactions. The system is based on the rate law of chemical reactions and considers entire biological system as chemical process. Optimizing the S-system variables is a crucial step in the gene network reconstruction. There are several bio-inspired algorithms used for the optimization purpose. Based on the evolution, the standard proposals are (Noman and Iba 2005; Huang et al. 2008; Mondal et al. 2010; Spieth et al. 2004; Kabir et al. 2008). Most of them are based on Genetic Algorithm theory. Apart from the evolution algorithms, there are successful proposals employing Cuckoo search (Jereesh and Govindan 2013a, b), particle swarm approach (Xu et al. 2007; Hsiao and Lee 2007; Yang et al. 2013) and artificial immune approaches (Jereesh and Govindan 2013c, 2014).

There are many bio-inspired algorithms to solve the reconstruction problem of gene regulatory network. The convergence speed of heuristic algorithms is a serious issue because they are highly nonlinear. High computational complexity and low accuracy are the main issues that need to be tackled. The tradeoff between time and accuracy factors of the algorithms still poses challenges. A systematic study will not end in itself because the biological systems are very sophisticated and the processes are highly nonlinear. Any addition of new knowledge would thus lead to an incremental understanding of the natural processes from a scientific viewpoint. So, there is ample scope for improvements in science and technology in the future. This paper proposes a novel and innovative technique to enhance the performance of the gene related process representations and understanding. The methodology followed is network modeling and verification through computational techniques using gene expression.

## Representation of gene network

Gene regulatory network generally can be represented as a graph *G*(*V*, *E*) in which vertices represent the gene and edges represent the relationship between them. The relationship between each gene represents overall performance of the gene network. A function or a set of functions determines the expression rate of each gene at a particular condition as described in Eq. (1).

1 $$F = \{ f_{i} |i \in {\mathcal{N}}\quad {\text{and}}\quad 1 \le i \le n\} ,$$

where, *n* is the total number of genes participating in the gene network, and *f* is a mathematical function whose inputs are current states (*X*(*t*) = *x*_1_(*t*),…, *x*_*n*_(*t*)) of the genes and output is next state of genes. The updated concentration, *x*_*i*_ of each gene *i* for time *t* + 1 is defined as Eq. (2).

2 $$x_{i} \left( {t + 1} \right) = f_{i} \left( {x_{1} \left( t \right),x_{2} \left( t \right), \ldots ,x_{n} \left( t \right)} \right).$$

The main features used for the pattern identification of gene network are the inhibitory relations and excitatory relations between genes. Inhibitory relation represents the genes capability to reduce the concentration of other genes and excitatory relation represents the opposite actions. In this context, a gene can act as both inhibitory and excitatory agent for another gene. There is positive inhibition and negative inhibition. Positive inhibition represents how gene increases the rate of inhibition. Negative inhibition represents how gene decreases the inhibition rate. Similarly, there is positive and negative excitation values depending on the behavior of the gene. Thus, there can be the following possibilities for the gene relations.

- A gene can act as inhibitory for one gene and excitatory for another gene.
- A gene can act as both inhibitory and excitatory for the other gene.
- At a time, a gene cannot act as positive and negative inhibitor.
- At a time, a gene cannot act as positive and negative excitatory.

The major decision variables for the gene network depend on the above-mentioned properties.

## Evaluation measures

In the literature, various standard methods are used to evaluate the results in the area being pursued. Most of the literature provides evaluation based on the comparison between their research findings and the relations identified by the biologists. It is very difficult to evaluate the research findings since the problem is to find the relationships in the biological system. There is a chance that the identified relations through biological experiments are incomplete. In such situations, to evaluate properly, there is a need for artificially generated data. Artificially generated data are those that generated by the models of biological system. With the combination of biological real life data and artificial data, we evaluated performance of various proposals in the thesis. The output microarray values for the biological system and artificially simulated system are categorized into accepted values and the values provided by the models proposed are categorized as the computed values.

For the evaluation, computed values have been compared with the accepted values. In the thesis, two types of comparison are performed for the evaluation. The first one focuses on the comparison of relations accepted by biologists and that computed by models. TP (true positive), FN (false negative), TN (true negative) and FP (false positive) are used for the comparison based on the biological relations. The second is based on the mean squared error (MSE) computed between accepted data values (that are generated biologically or artificially) and computed microarray data values.

The important metrics used to evaluate the gene regulatory network reconstruction in literature are briefly presented in the following subsections.

### Fitness function

Mean squared error (MSE) proposed by the Tominaga et al. (2000) is used as the fitness function for the optimization.

3 $$f = \mathop \sum \limits_{i = 1}^{N} \mathop \sum \limits_{t = 1}^{T} \left( {\frac{{x_{i,t}^{\text{cal}} - x_{i,t}^{ \exp } }}{{x_{i,t}^{{ {\text{exp}}}} }}} \right)^{2} ,$$

where $x_{i,t}^{\exp }$, $x_{i,t}^{\text{cal}}$ are the expression value of gene *i* at time *t* from the experimental and estimated (calculated) data, respectively. Here, *N* is the total number of genes and *T* is the time interval.

### Sensitivity

This measurement calculates the fraction of accepted relations that are identified. Equation for the sensitivity is given as expression (4).

4 $${\text{Sensitivity}} = \frac{\text{TP}}{{{\text{TP}} + {\text{FN}}}}{ \times }100,$$

where TP is the true positive, that is, number of accepted relations identified, FN is the false negative, that is, number of accepted relations that are not identified. The percent sensitivity value is 100 if all the accepted relations are identified.

### Specificity

This measurement calculates the fraction of unaccepted biological relations that were shown unaccepted. The equation for evaluating specificity is given as expression (5).

5 $${\text{Specificity}} = \frac{\text{TN}}{{{\text{TN}} + {\text{FP}}}}{ \times }100 ,$$

where TN is the true negative, that is, number of unaccepted relations shown unaccepted, FP is the false positive, that is, number of unaccepted relations that were identified as accepted. The specificity percent value is 100 if all the unaccepted relations are identified as unaccepted.

### Balanced accuracy

Data related to bioinformatics is inadequate and imbalanced, hence, to measure the accuracy of the research, in the problem mentioned, a parameter called balanced accuracy is used. The RHS of Eq. (6) represents the expression for balanced accuracy. It is the mean of the sensitivity and specificity measures computed as follows.

6 $${\text{Balanced accuracy}} = \left( {\frac{{{\text{Sensitivity}} + {\text{Specificity}}}}{2}} \right){ \times }100$$

Similar to all percentage problems, if the balanced accuracy increases the result will be more similar to reality and the values would lie between 0 and 100, 100 being close to reality.

## S-system model

Most popular differential equation model is the S-system model, which is a well-accepted nonlinear differential equation modeling proposed by Savageau (Savageau 2000). In the S-system, the rate of change of concentration of gene xi is defined as in Eq. (7).

7 $$\frac{{{\text{d}}x_{i} }}{{{\text{d}}t}} = \alpha_{i} \mathop \prod \limits_{j = 1}^{N} x_{j} \left( t \right)^{{G_{i,j} }} - \beta_{i} \mathop \prod \limits_{j = 1}^{N} x_{j} \left( t \right)^{{H_{i,j} }} ,$$

where *G*_*i,j*_ and *H*_*i,j*_ are excitatory and inhibitory coefficients, respectively. *α*_*i*_ ≥ 0 and *β*_*i*_ ≥ 0 are rate constants. *G*_*i,j*_ and *H*_*i,j*_ represent the relationship between each genes and *x*_*j*_(*t*) is the concentration of gene *j* expressed at time *t*.

S-system is a power law formalism, which is inspired from the chemical reaction processes. According to the rate law, the rate of change of concentration of each reactant is represented by the equation *k*(*T*)[*C*_*A*_]^*x*^[*C*_*B*_]^*y*^, where *k*(*T*) is the rate constant which is having a dependency with the temperature *T*. *C*_*A*_ and *C*_*B*_ express the concentration of the species *A* and *B*. The exponents *x* and *y* are reaction orders. Gene regulation interaction is a bio-chemical process that takes place in a living organism. This model demonstrates the chemical reaction that happens between genes. The total number of variables used in the S-system is 2*N* + 2*N* ^2^, where *N* is the number of genes in the reaction.

In this paper, S-system is the mathematical model used to model the GRN.

## Immuno-hybrid based S-system model computation

**Fig. 1:**
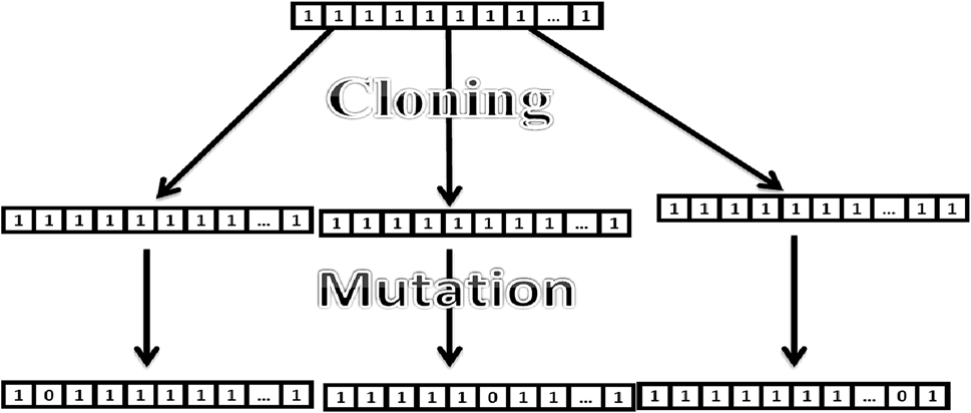
Mutated cloning method

This algorithm is a global–local optimization approach to solve the problem of gene regulatory network reconstruction using the DNA microarray data. This approach is a combination of clonal selection algorithm (Jereesh and Govindan 2013c) and BFGS Quasi-Newton algorithm (Dennis and More 1977). Clonal selection algorithm is a meta-heuristic algorithm used for the optimization problems. In the clonal selection algorithm maturation step is replaced with two operations. First one is the cloned mutation process, in which each clone of the selected antibodies are mutated using a mutation probability *m*. Diagrammatic representation of general cloned mutation process is depicted as in Fig. 1. Second one is the local weight updating process, in which each mutated antibody is updated using the BFGS Quasi-Newton method. In the second step, dynamic step size change is introduced as per the Eq. (8). Each antibody holds inherited properties from the parent and the divergence properties due to mutation. Detailed algorithm for the immuno-hybrid approach is given as Algorithm 1.

**Fig. 2:**
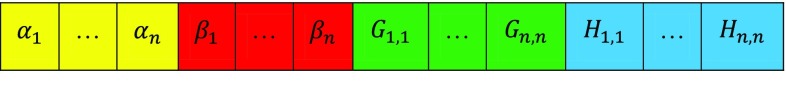
Format of the vector representing an individual cuckoo





For the gene regulatory network construction, each antibody in the population stands for the parameters of the S-system. Identifying the optimal antibody solution is the problem specified here. Format of the vector representing individual antibody is given in Fig. 2. Fitness evaluation is done using Eq. (3). For fitness calculation, at first the individual antibody is modeled as a vector representing the solution parameters of the S-system model. Then, the differential equation of S-system is solved by Runge–kutta algorithm. Original microarray values and generated microarray values are used to compute fitness value using Eq. (3).

8 $$\mu = {\text{round}}\left( {\lambda { \times }\left( {P - R_{\text{f}} - 1} \right)} \right),$$

where *μ* is the maturation rate, *λ* is the local iteration factor; *P* is the population size and *R*_f_ is the rank of individual based on the fitness value.

BFGS Quasi-Newton formula

9 $$B_{k + 1} = \, B_{k} + \, (r_{k} r_{k}^{T} / \, r_{k}^{T} \delta_{k} ) - (B_{k} \delta_{k} \delta_{k}^{T} B_{k} /\delta_{k}^{T} B_{k} \delta_{k} ),$$

where *B*_*k*_ is an approximate hessian matrix,

10 $$s_{k} = \alpha_{k} d_{k}$$

11 $$y_{k} = \, g_{k + 1} - \, g_{k}$$

## Results and discussions

For comparing the efficiency of the proposed approaches in the paper, a well-known five-gene standard artificial network (Noman and Iba 2005; Kimura et al. 2008; Kabir et al. 2008) is identified. The simulated microarray data is generated using the Runge–kutta algorithm and S-system model. For the experimentation, ten sets of expression data with initial values in the interval [0, 1] are generated artificially.

**Fig. 3:**
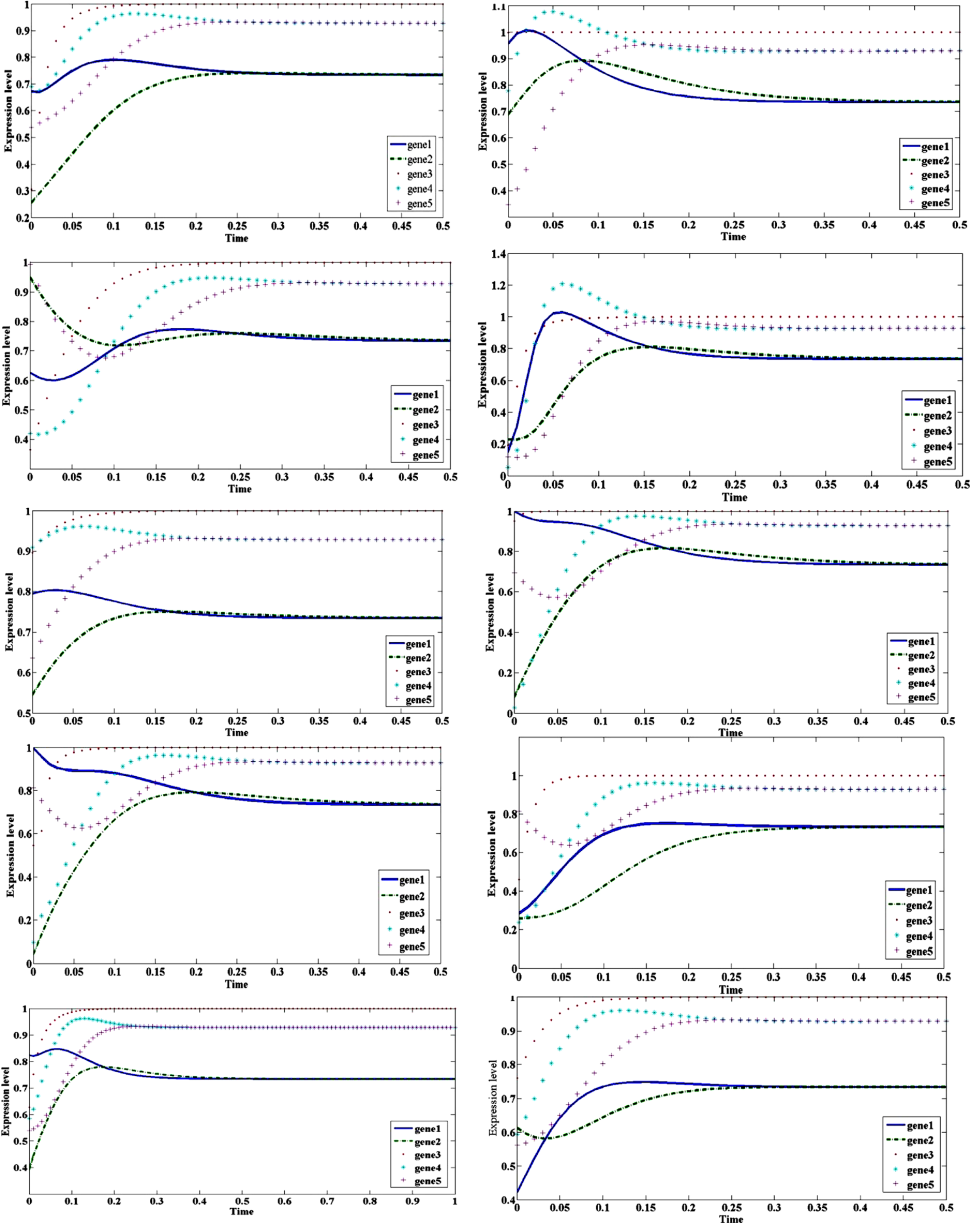
Time-dynamics of the ten data sets of five-dimensional regulatory system

The initial values used for the generation of artificial data are taken as per (Jereesh and Govindan 2013c). To average the performance over more data sets, we generated ten artificial data sets with the help of a model by Hlavacek and Savageau (1996) and the time dynamics of data sets are given in Fig. 3.

**Fig. 4:**
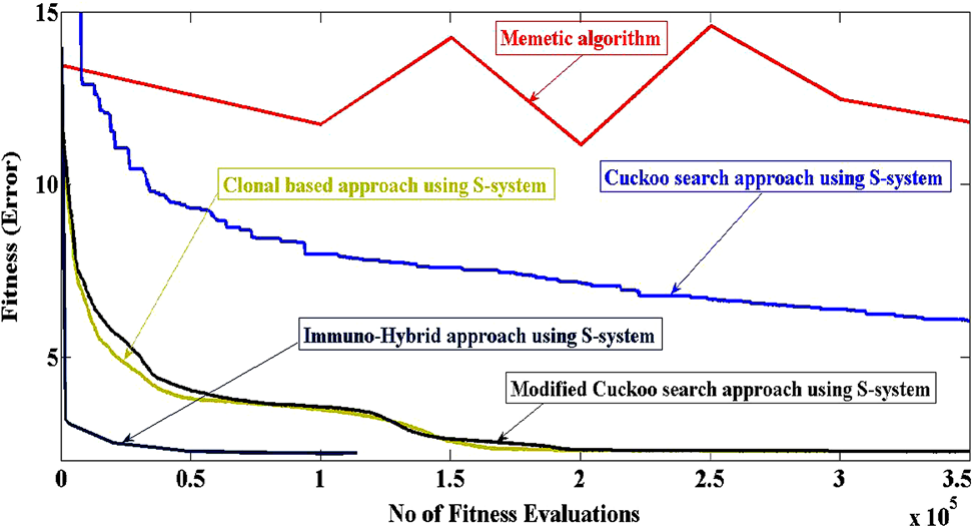
Performance of convergence. Comparison of errors obtained for memetic algorithm, cuckoo search using S-system, modified cuckoo search using S-system, clonal based approach using S-system model and immuno-hybrid approach using S-system

The proposed immuno-hybrid approach for optimizing the parameters of S-system is compared with the other approaches (Spieth et al. 2004; Jereesh and Govindan 2013a, b, c) in literature. For the artificial data, performance comparison with respect to the convergence is depicted in Fig. 4. It is evident that the immuno-hybrid approach outperforms all of the mentioned approaches. The immuno-hybrid approach using S-system converged after 0.9 × 10^5^ fitness evaluations, which is a good improvement in speed performance, compared to other algorithms.

### SOS DNA repairing system in *E. coli*: real world experimental data

*Escherichia coli* (*E. coli*) are bacterium residing in the lower intestine of warm-blooded organisms. This causes food poisoning occasionally. Most of the *E. coli* is harmless for the host and some will help to produce vitamin K2. This also helps for the proper digestion of the food. This bacterium is easy to reproduce within a time limit on laboratory conditions and important species in the study of molecular biology.

**Fig. 5:**
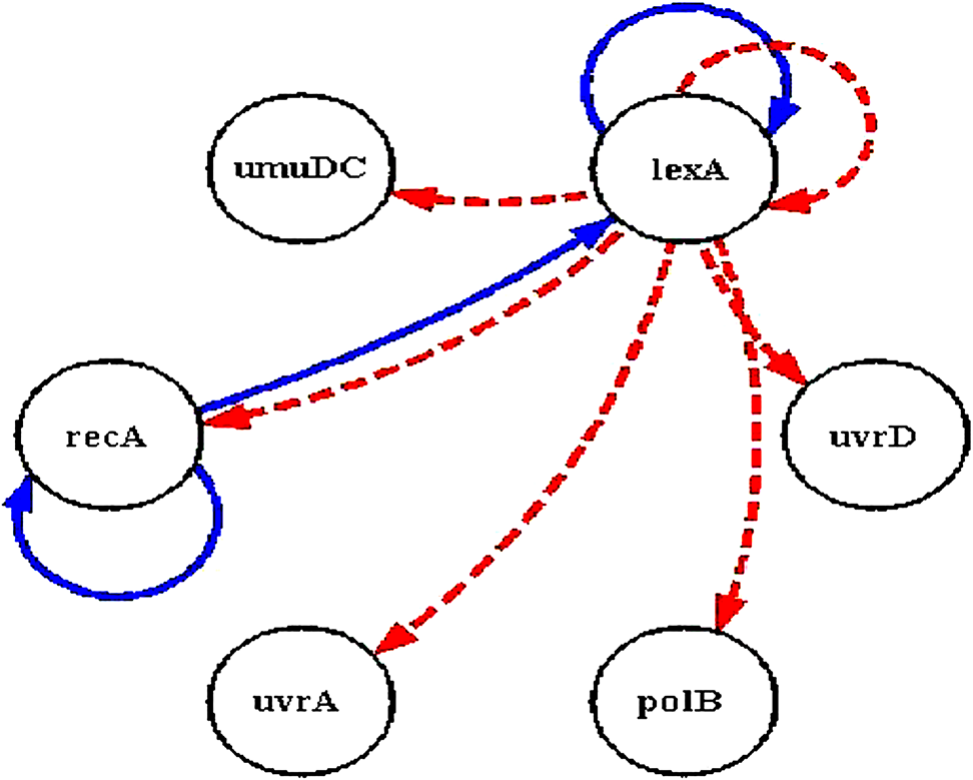
SOS DNA repair system of *E. coli* (*solid lines* indicate the activation and *dashed lines* indicate the inhibition)

SOS DNA repair system in *E. coli* (Sutton et al. 2000) is a famous real life data set, which is commonly used to evaluate the efficiency of gene regulatory network reconstruction methods. Figure 5 is a graphical representation of gene interactions following the damage of DNA. Mainly six major genes (*uvrD*, *lexA*, *umuD*, *recA*, *polB* and *uvrA*) are involved in the processing of DNA repair (Kimura et al. 2008, 2009; Kabir et al. 2008; Hsiao and Lee 2007). *LexA* is a repressor gene, which inhibits the expression of other genes. Whenever DNA damage happens in *E. coli*, *RecA* identifies the damage and activates the processing of cleavage of *LexA*. Hence, the concentration of the *LexA* is reduced and leads to the excitation of other genes. After the clearance of damage, cleavage of *LexA* will be slow downed and stopped, and this leads to increased concentration of *LexA*. The *LexA* represses the other genes and advances to a balanced state. Construction of gene network allows predicting the roles of each of the genes in the DNA repairing system.

SOS DNA repair system of *E. coli* data set is obtained from the experiments done by Uri Alon lab of Weizmann Institute of science; (website http://wws.weizmann.ac.il/mcb/UriAlon/download/downloadable-data). There are 50 time-periods for the experiment in which 49 are used for the experimentation where the first time-period is at zeroth time and contains zero knowledge. Out of the eight genes we have selected, six important genes are specified. All the values in the expression have been normalized in the range of [0, 1].

**Fig. 6:**
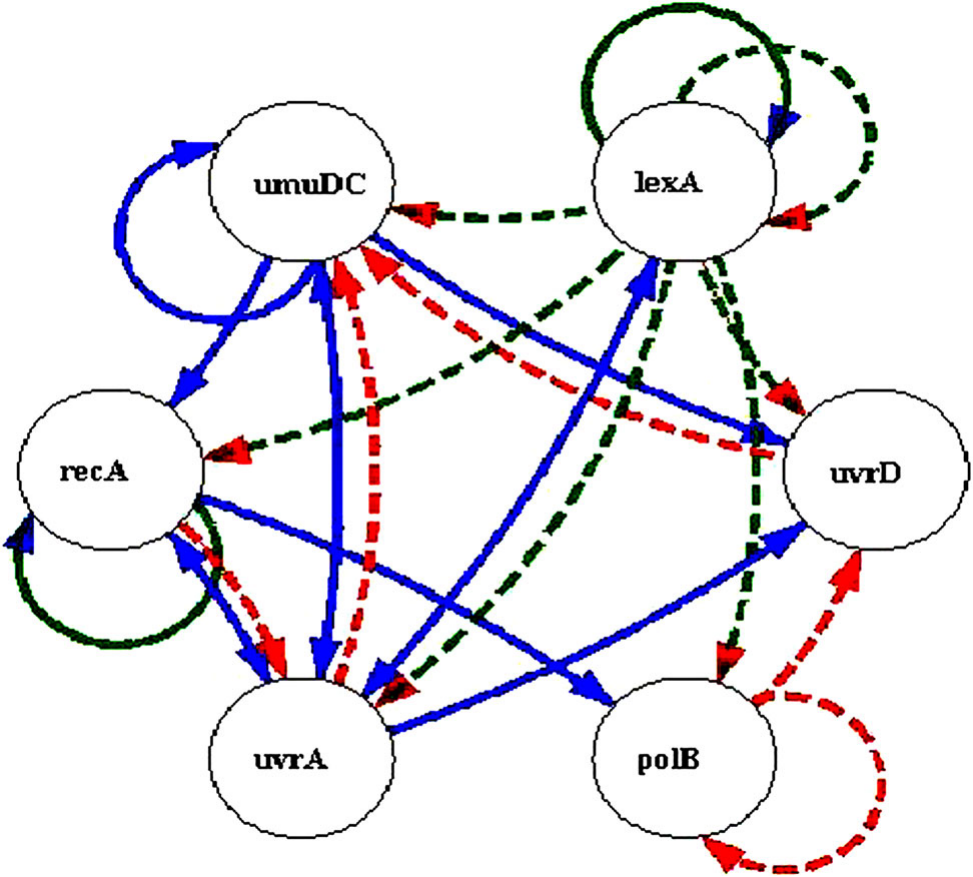
SOS DNA repair system of *E. coli* identified immuno-hybrid based approach using S-system model (*dotted line* indicates the inhibition and *solid line* indicates the activation, *green lines* indicate the relations that were also identified by the biologists)

Gene regulatory network for DNA repair system of *E. coli* identified by the immuno-hybrid approach is depicted as in Fig. 6. The immuno-hybrid based approach using S-system model identified eight relations, which was identified by the biologists (Table 1).

**Table 1:** Comparison of number of relations identified by the proposed approaches with other approaches in the literature for SOS DNA repair system of *E. coli*

Gene relations	Huang et al. (2008)	Hsiao and Lee (2007)	Mondal et al. (2010)	d’Alché-Buc et al. (2005)	Kimura et al. (2009)	Noman and Iba (2005)	Kimura et al. (2008)	Noman et al. (2013)	Jereesh and Govindan (2013a)	Jereesh and Govindan (2013c)	Kabir et al. (2008)	Jereesh and Govindan (2013b)	Yang et al. (2013)	Immuno-hybrid S -system
*LexA* -*|RecA*	×	×	√	√	√	√	√	√	√	√	√	√	√	√
*LexA* -*|LexA*	×	√	√	√	√	√	√	×	√	√	√	√	√	√
*LexA* - > *LexA*	×	×	×	×	×	×	×	×	√	×	×	√	×	√
*LexA* -*|umuDc*	√	×	×	×	√	√	√	√	×	×	√	√	×	√
*LexA* -*| uvrA*	×	√	√	√	×	√	×	√	√	√	√	√	√	√
*LexA* -*| uvrD*	×	√	√	×	√	×	√	√	×	√	√	×	√	√
*LexA* -*| polB*	×	×	×	×	√	×	√	√	√	√	√	×	√	√
*RecA* - > *LexA*	√	×	×	√	×	√	×	×	√	×	×	√	√	×
*RecA* - > *RecA*	×	×	×	×	×	×	×	×	×	√	×	√	×	√
No of relations identified correctly	2	3	4	4	5	5	5	5	6	6	6	7	6	8
Sensitivity (%)	22	33	44	44	56	56	56	56	67	67	67	67	66.6	88.9
Specificity (%)	35	69	64	72	61	69	81	47	52	15	48	61	73.9	52.2
Balanced Acc (%)	28.5	51	54	58	58.5	62.5	68.5	51.5	59.5	41	57.5	64	70.2	70.55

## Conclusions

Biological systems behave differently in different conditions. To model such systems we need a dynamical modeling. A nonlinear differential equation modeling for the dynamic biological systems is a common approach. This paper proposed an evolutionary global–local hybrid algorithm for the optimization of gene regulatory network modeling. An algorithm called clonal selection based optimization algorithm is combined with the BFGS Quasi-Newton search method to develop immuno-hybrid algorithm. The randomness property, cloned mutated process and local weight updating property are the key factors for the immuno-hybrid approach. The proposed approaches predicted gene network for artificial data set and SOS DNA repair system more efficiently than many of the existing algorithms. The convergence speed and accuracy compared with the existing approaches are found to be superior. The immuno-hybrid approach provided still better performance figures of 89 and 70.5, respectively, for sensitivity and balanced accuracy. In addition, immune-hybrid approach identified eight valid relations, which is the highest among all of the other algorithms. Thus, it is demonstrated that a combination of differential modeling and hybrid optimization techniques can provide better reconstruction of gene network.
